# Immune Checkpoints: Therapeutic Targets for Pituitary Tumors

**DOI:** 10.1155/2021/5300381

**Published:** 2021-08-16

**Authors:** Ding Nie, Yimeng Xue, Qiuyue Fang, Jianhua Cheng, Bin Li, Dawei Wang, Chuzhong Li, Songbai Gui, Yazhuo Zhang, Peng Zhao

**Affiliations:** ^1^Department of Neurosurgery, Beijing Tiantan Hospital, Capital Medical University, Beijing, China; ^2^Savaid Medical School, University of Chinese Academy of Sciences, Beijing, China; ^3^Beijing Neurosurgical Institute, Beijing, China

## Abstract

Pituitary tumors are the third most common intracranial tumors in adults. Treatment of refractory pituitary tumors is known to be difficult due to limited treatment options. As a promising therapeutic method, tumor immunotherapy has been applied in the treatment of many tumors, including pituitary tumors. Immune checkpoint blocking is one of the effective strategies to activate antitumor immunity. Immune checkpoints prevent tissue damage by regulating the immune response of peripheral tissues and participate in the maintenance of a normal immune environment. In the presence of a tumor, inhibition of T cell activity by tumor cells binding to immune checkpoints and their ligands is an important mechanism for tumor cells to escape immune injury. In this review, we summarize the latest findings of immune checkpoints and their potential as immunotherapeutic targets for pituitary tumors.

## 1. Introduction

**Figure 1:**
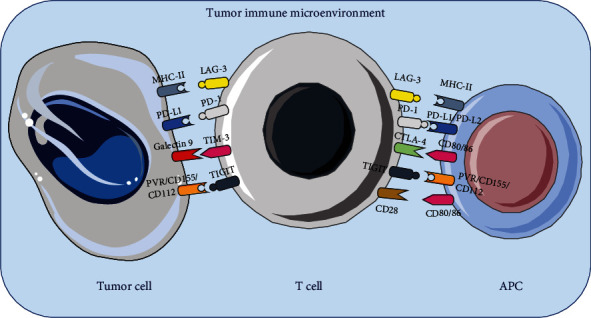
Binding patterns of immune checkpoints. Immune checkpoint binding with ligand in the immune microenvironment.

A pituitary tumor is a nonmetastatic tumor that occurs in the pituitary gland and accounts for 15% of all tumors of the central nervous system [1, 2]. A small proportion of pituitary tumors are clinically invasive and are likely to remain or recrudesce after surgery and radiotherapy [3]. Temozolomide (TMZ) is effective in some invasive pituitary tumors, but up to 50% of patients do not respond to TMZ, and the median time of progression is short [4]. Targeted therapies, including growth factors and their receptors, intracellular signaling pathways, and proteins that regulate cell cycles, are also of limited effectiveness [5]. At the same time, some pathologists have suggested that invasive pituitary tumors have malignant potential, and early identification and aggressive treatment of these invasive tumors are needed to reduce tumor recurrence and prolong survival [6]. Thus, it is urgent to propose a new treatment regimen. In recent years, based on the deep understanding of tumor immune microenvironment, immune checkpoint suppressive therapy has made great progress in cancer treatment which applied to the treatment of various malignant tumors including melanoma, lymphoma, lung cancer, bladder cancer, liver cancer, and gastroesophageal cancer [7–9]. Different immune checkpoints work together to regulate the immune system, which is a double-edged sword, and in physiological situations, these checkpoints are usually responsible for maintaining the immune response within the required physiological range and protecting the host from autoimmunity. In the presence of a tumor, immune checkpoints may be used to inhibit the activation of T cells, thereby preventing T cells from damaging tumor cells and eventually leading to tumor proliferation or migration [10–12] (Figure 1). Thus, targeted immune checkpoint therapy is a new hot spot in tumor immunotherapy. Immune checkpoint inhibitors (ICIs) mediated immunotherapy has become a turning point in oncology therapy by targeting immune checkpoints, relieving T cell suppression, and promoting antitumor immunity [13, 14]. So far, the cytotoxic T-lymphocyte antigen, 4 (CTLA-4) and programmed cell death protein 1 (PD-1)/programmed cell death ligand 1 (PD-L1) are the main representative immune checkpoints. Meanwhile, Lymphocyte activation-gene-3 (LAG-3), T cell immunoglobulin domain and mucin domain-3 (TIM-3), and T cell immunoreceptor with Ig and Itim domains (TIGIT) have been identified (Table 1) [13, 15, 16]. In the case of pituitary tumors, with the further study of its immune microenvironment, the use of immune checkpoint inhibitors may be the next effective choice for the treatment of refractory pituitary tumors or even pituitary cancer. [10]. Therefore, this review article introduces the current research progress of different immune checkpoints and discusses their application prospects in pituitary tumors.

## 2. PD-1/PD-L1

PD-1 is often expressed on the surface of B cells, T cells, NK cells, and other cells, and the combination with PD-L1 and PD-L2 will block the cytokine secretion and proliferation of these cells [17, 18]. Although PD-L2 can also inhibit T cell function, PD-1/PD-L1 blockers have received more attention because PD-L1 expression is higher in tumor cells than PD-L2, that is, in many human tumors, the high expression of PD-L1 leads to poor prognosis [19–22]. PD-1/PD-L1 axis inhibitors exert their antitumor effects by alleviating PD-L1-mediated inhibition of tumor-infiltrating T lymphocytes and enhancing the proliferation of tumor-infiltrating T-regulatory cells (Treg) [23, 24]. For example, inhibitors block the interaction of PD-1 receptors on CD8^+^ and CD4^+^ T cells with PD-L1 on target tumor cells [25, 26]. The expression of PD-L1 is a predictive biomarker of anti-PD-1/PD-L1 treatment response. It has been reported that in different tumor types, PD-L1-positive patients with tumors have a much higher response rate to PD-1/PD-L1 axis inhibitors than the negative ones [27]. Several studies have described the expression of PD-L1 in pituitary neuroendocrine tumors (PitNETs). In general, the present studies indicated that PD-L1 is highly expressed in invasive pituitary tumors as well as in some functional pituitary tumors, particularly in somatotrophs and lactotrophs [28–33]. Furthermore, in a recent study of 264 pituitary adenoma specimens, researchers found a high incidence of significant overexpression of PD-L1 in Pit-1-positive tumors [34]. Compared to tumor tissues, a study involving 10 pituitary samples showed no increase in PD-L1 expression in normal endocrine tissues [35]. Although some types of pituitary tumors show high levels of PD-L1 expression, it does not mean that these tumors will necessarily respond to immune checkpoint suppression. Still, these facts suggest that immune checkpoint suppression may represent a reasonable treatment for some pituitary tumors and even pituitary carcinomas [34]. Likewise, pituitary tumors themselves exhibit T cell infiltrates, a prerequisite for checkpoint blockade efficacy [28]. In preclinical studies, after subcutaneous tumor implantation, anti-PD-L1 treatment significantly inhibited tumor growth and serum ACTH secretion, and some mice achieved complete tumor regression, compared with tumor-bearing mice without anti-PD-L1 treatment, which also have been observed in models of intracranial tumors [32]. There has been strong evidence of the effectiveness of immunotherapy in the treatment of pituitary tumors. In 2018, Lin et al. reported about a patient with ACTH-secreting pituitary carcinomas who was successfully treated with combined immunotherapy with ipilimumab (anti-CTLA-4) and nivolumab (anti-PD-1) [36]. Recently, Sol et al. reported about a patient with ACTH-secreting pituitary carcinomas who was stabilized with the same combination immunotherapy [37]. Caccese et al. also reported about a patient with a MMRd pituitary adrenocorticotropic hormone- (ACTH-) secreting adenoma treated with the checkpoint inhibitor pembrolizumab [38]. Similarly, Lamb et al. treated a case of prolactin pituitary cancer using ipilimumab and nivolumab in combination with vascular endothelial growth factor inhibition therapy [39]. In conclusion, the exploration of immunotherapy in pituitary tumors or pituitary carcinomas with high PD-L1 expression is a promising work.

## 3. CTLA-4

CTLA-4 is a kind of representative immune checkpoint pathway like PD-1/PD-L1 and is also involved in the negative regulation of immune function at different stages of T cell activation [13]. CTLA-4, expressed on activated T and Treg cells, is homologous to CD28 and has a higher affinity for CD80 and CD86 [40]. Unlike the first antigen-dependent receptor (CD28), CTLA-4 is antigen independent [41]. It is the second receptor of the T cell costimulatory ligand CD80/86, and its function is critical for the downregulation of the immune response. Typically, CD28 binds to the B7 ligand and signals through phosphoinositol 3-kinase (PI3K) to enhance downstream activation pathways [42]. The binding of CTLA-4 to CD80/CD86 prevents T cell proliferation stimulation provided by the binding of CD28 to CD80/CD86 during initiation [40]. In addition, the involvement of CTLA-4 in T cell activation prevents cell cycle progression [43]. In animal experiments, Brunner et al. demonstrated that CTLA-4 prevented cell cycle progression by inhibiting the production of cyclin D3 and CDK4 and CDK6, as well as altering the degradation of cell cycle inhibitor p27. Moreover, they also observed CTLA-4-mediated effects on cyclins when cells were stimulated only by CD3, suggesting that CTLA-4 inhibited the CD28-independent pathway in T cell activation [44]. CTLA-4 blocks the binding of antibodies to CTLA-4 expressed on T lymphocytes, leading to the beneficial expansion of effector T cells that recognize tumor antigens and eliminate tumors, thereby inhibiting tumor growth [45]. Currently, CTLA-4 has been rarely reported in pituitary tumors. In one study, the transcriptome of 115 pituitary tumors was analyzed and no differences in CTLA-4 expression among tumor subtypes were observed which showed that the expression of CTLA-4 was not specific in each subtype of pituitary tumor [31]. In another study, CTLA-4 expression was confirmed in 37 surgical pituitary adenomas and 11 normal pituitary glands [45]. These shreds of evidence explain that the application of CTLA-4 antibodies binds to the pituitary CTLA-4 and triggers a series of cytopathic immune responses leading to side effects such as pituitary inflammation [46]. At the same time, it is also a kind of evidence of the therapeutic effect of CTLA-4 antibody on pituitary tumors. Considering that the combination of CTLA-4 antibody with the “ectopic” expression of CTLA-4 antigen on normal pituitary endocrine cells can cause damage to normal pituitary tissue, the use of the CTLA-4 antibody as an adjuvant to other checkpoint inhibitors, such as anti-PD-1 and anti-PD-L1, for the treatment of pituitary tumors, may be a promising approach. CTLA-4 inhibitors are used in combination with PD-1/PD-L1 inhibitors in the treatment of pituitary tumors reported so far [36].

## 4. TIM-3, LAG-3, and TIGIT

The success of CTLA-4 inhibitors and PD-1/PD-L1 inhibitors in the treatment of many types of tumors has inspired researchers to investigate targets beyond these [47]. Although to the best of our knowledge, no studies have targeted these targets in pituitary tumors, they remain a viable and promising option.

TIM-3, a member of the TIM gene family, is expressed in tumor cells and immune cells [48–50]. The interaction of TIM-3 with its ligand induces T cell inhibition, while blocking TIM-3 expression leads to T cell proliferation and cytokine production, thus triggering immune activation [51]. Notably, Tim-3 and PD-1 are often coexpressed in T cells, which are dysfunctional or failing. In one study, anti-TIM-3 treatment alone had little or no effect on mice carrying solid tumor CT26 colon cancer, and anti-PD-L1 treatment alone showed a tendency to delay tumor growth [48]. However, the combination of anti-TIM-3 and anti-PD-L1 led to a significant reduction in tumor growth, with 50% of the mice showing complete tumor regression [48]. This suggests that combined targeting of the TIM-3 and PD-1 pathways is more effective in controlling tumor growth than targeting the TIM-3 and PD-1 pathways alone. Song et al. reported that Tim-3^+^ Foxp3^+^ Treg cell levels in PBMC of patients with nonfunctional pituitary adenoma were significantly higher than those of healthy controls, and the level of Foxp3^+^ Treg cells expressing Tim-3 was significantly reduced in patients after surgery [52]. Therefore, it is worth further exploration whether combined blocking of TIM-3 and PD-L1 can effectively treat pituitary tumors.

LAG-3 is expressed in activated CD4^+^ and CD8^+^ T cells, NK cells, B cells, and dendritic cells (DC) and induces immune failure by binding to major histocompatibility complex class II (MHC-II) and other ligands [53–56]. Coexpression of LAG-3 with other targets (such as PD-1, TIGIT, and TIM-3) leads to T cell failure, exemplified by lack of proliferation and cytokine secretion [57]. In some preclinical studies on different tumor types, LAG-3 monotherapy has largely failed, often in combination with other targets, i.e., the use of dual coblocking will enhance tumor inhibition [58–60]. For example, in mice with MC38 tumor, dual LAG-3/PD-1 coblocking synergism restricted the growth of MC38 and resulted in 80% tumor clearance in mice [57]. It seems that coblocking of LAG-3 with PD-1 or other targets can enhance the effect of immunotherapy, and whether it can also be applied in pituitary tumors is a direction of future research.

TIGIT expression in NK cells and T cells, binding with CD155, CD112, or PVRL3, can inhibit interferon-*γ* production of NK cells and promote the generation of mature immunoregulatory DCS and inhibitory differentiation and function of T cells [47, 61–63]. Similar to TIM-3 and LAG-3, TIGIT is often coexpressed with other targets, and double blockade restates T cell and NK cell function in the preclinical environment [62, 64, 65]. Studies have shown that activation of the hypothalamic-pituitary-adrenal (HPA) axis causes the adrenal cortex to release glucocorticoid (GC) hormones into the circulatory system, and these GCs may promote the expression of TIGIT by key effector cells in an environmental, tissue-specific, and system-specific manner, thereby suppressing the immune response [66]. Whether the secretion of hormones in functional pituitary tumors can also affect the expression of TIGIT and the coinhibition of TIGIT and other targets can affect the immunotherapy of pituitary tumors are two problems facing us at present.

## 5. The Dilemma of Immune Checkpoint Inhibitors

The use of immune checkpoint inhibitors has revolutionized the management of some tumors, but with different responses in different patients, and these drugs can cause unique adverse reactions that can be life threatening [67–69]. How to enhance the efficacy of immunosuppressive agents and reduce adverse reactions are two major challenges facing us.

The combination of immunosuppressive agents, such as CTLA-4 inhibitors combined with PD-1 inhibitors, or TIM-3 and PD-L1 inhibitors combined with blocking, is currently the best choice to enhance the therapeutic effect [17, 36, 48]. Some researchers are exploring other ways, too. For example, Deng et al. proposed that the combination of radiotherapy and anti-PD-L1 therapy can enhance the activity of CD8^+^ T cells, optimize the tumor immune microenvironment, and lead to tumor regression [70]. Liu et al. believed that abnormal mechanical properties and immunosuppression were the two key factors limiting the antitumor efficacy of T cell immune checkpoint blocking inhibitors against solid tumors in clinical practice, and they found that hyperbaric oxygen could promote PD-1 antibody delivery and destroy hypoxic-mediated immunosuppression through the consumption of extracellular matrix [71]. Melero et al. suggested that intratumoral drug delivery and targeted drug delivery of tumor tissue should be used instead of traditional intravenous infusion [72]. Such explorations provide broad ideas for finding ways to improve the efficacy of immunosuppressants.

In clinical treatment, immune-related adverse events (irAE) caused by ICIs are more toxic than conventional chemotherapy and often involve different organ systems [73, 74]. For example, the cardiovascular system presents with pericarditis, pericardial effusion, and various types of arrhythmias, including the development of the complete atrioventricular block, myocardial infarction, heart failure, and myocarditis [75]. In the skin lesions, the manifestations are psoriasis, erythema pleomorphic, leukocyte cataclastic vasculitis, and eczema [76]. In the gastrointestinal system, diseases such as colitis, hepatitis, cholangitis, and gastritis are common [77]. In the blood system, it is often manifested as autoimmune hemolytic anemia, immune thrombocytopenia, and aplastic anemia [78]. In the nervous system, it is manifested as myasthenia gravis, encephalitis, and demyelination of the central nervous system [79]. In the urinary system, acute kidney injury and acute tubulointerstitial nephritis often occur [73]. When considering the introduction of ICIs in the treatment of pituitary tumors, it is of concern that ICIs can damage the endocrine system and cause endocrine diseases involving the thyroid, pituitary, adrenal, and pancreas [80]. How to reduce the damage to normal pituitary tissue while making ICI damage to pituitary tumor cells is the key to make immune checkpoint suppression therapy suitable for a pituitary tumor.

## 6. Future Perspectives and Conclusions

While ICIs have been extensively used to target immune checkpoints in many tumors, their use in pituitary tumors has just commenced. With the increasing research on the microenvironment of pituitary tumors, the recognized infiltration of lymphocytes and the expression of immune checkpoints seem to give us a strong implication that immunotherapy targeting immune checkpoints is the next effective treatment approach for pituitary tumors. Before that, to clarify the specific mechanism of the interaction between pituitary tumors and the human immune system, improve the efficacy of ICIs, and reduce irAE are the first three problems to solve. There are reasons to believe that immune checkpoints will be the next therapeutic target for pituitary tumors.

## Figures and Tables

**Table 1:** Possible immune checkpoints in pituitary tumors.

Immune checkpoint	Application in pituitary tumors	Research type	Recommendation	Ref.	Year
PD-1/PD-L1	Cushing's disease ACTH pituitary carcinomas Prolactin pituitary carcinomas	Preclinical Clinical Clinical	Used in pituitary tumors with high expression of PD-L1, combined with other target inhibitors when necessary	[32] [37] [38] [39]	2020 2021 2020 2020
CTLA-4	ACTH pituitary carcinomas Prolactin pituitary carcinomas	Clinical Clinical	Combination therapy of CTLA-4 inhibitors with PD-1/PD-L1 inhibitors	[37] [39]	2021 2020
TIM-3	—	—	Tim-3 blocking combined with PD-L1 blocking	—	—
LAG-3	—	—	Combined with other targets, dual blocking	—	—
TIGIT	—	—	Combined with other targets, dual blocking (functional pituitary tumors)	—	—

PD-1/PD-L1: programmed cell death protein 1/programmed cell death ligand 1; CTLA-4: cytotoxic T-lymphocyte antigen, 4; TIM-3: T cell immunoglobulin domain and mucin domain-3; LAG-3: lymphocyte activation-gene-3; TIGIT: T cell immunoreceptor with Ig and Itim domains.
